# Methodology of the Updated and Expanded Australasian Bronchiolitis Guideline

**DOI:** 10.1111/jpc.70145

**Published:** 2025-07-17

**Authors:** Kate Loveys, Emma J. Tavender, Franz E. Babl, Elizabeth Cotterell, Libby Haskell, Sharon O’Brien, Ed Oakley, Catherine Wilson, Meredith L. Borland, Stuart R. Dalziel, the Paediatric Research in Emergency Departments International Collaborative (PREDICT) Network, Jane Alsweiler, David Armstrong, Simon Craig, Nigel Crawford, Dianne Crellin, Sonja Crone, Trevor Duke, Shane George, Christine Jeffries‐Stokes, Nidhi Krishnan, Anna Lithgow, Ken Peacock, Tomas Ratoni, Peter Richmond, Annie Smith, Rebecca Starkie, David Thomas, Alexandra Wallace, Michael Zhang, Poh Chua, Marian Chandler, Paul Bauert, Christine Brabyn, Lydia Garside, David Levitt, Nicola McKay, Jocelyn Neutze, Andreas Schibler, Kam Sinn, Janine Spencer, Helen Stevens

**Affiliations:** ^1^ Department of Paediatrics: Child and Youth Health, School of Medicine The University of Auckland Auckland New Zealand; ^2^ Emergency Research, Clinical Sciences Murdoch Children’s Research Institute Parkville Victoria Australia; ^3^ Departments of Paediatrics and Critical Care The University of Melbourne Parkville Victoria Australia; ^4^ Australian Catholic University Sydney New South Wales Australia; ^5^ Emergency Department Royal Children’s Hospital Parkville Victoria Australia; ^6^ Armidale Rural Referral Hospital Armidale New South Wales Australia; ^7^ School of Rural Medicine and Tablelands Clinical School University of New England Armidale New South Wales Australia; ^8^ Children’s Emergency Department Starship Children’s Hospital Auckland New Zealand; ^9^ Emergency Department Perth Children’s Hospital Perth Western Australia Australia; ^10^ Division of Emergency Medicine, Anaesthesia and Pain Medicine, Medical School The University of Western Australia Perth Western Australia Australia; ^11^ Institute for Paediatric Perioperative Excellence The University of Western Australia Perth Western Australia Australia; ^12^ Clinical Sciences Murdoch Children’s Research Institute Parkville Victoria Australia; ^13^ Department of Surgery, School of Medicine The University of Auckland Auckland New Zealand

**Keywords:** bronchiolitis, guideline methodology, infants, paediatric, respiratory

## Abstract

**Background:**

Bronchiolitis is the most common reason for hospital admission in infants in Australia and Aotearoa New Zealand (AoNZ), with care historically affected by practice variation, including use of ineffective therapies. The Paediatric Research in Emergency Departments International Collaborative (PREDICT) developed the first Australasian (Australia, AoNZ) bronchiolitis guideline in 2016, providing evidence‐based guidance on the management of infants (< 12 months of age), presenting or admitted to hospital with bronchiolitis. In 2022, PREDICT initiated a guideline update to include new evidence and expand the scope to include intensive care (up to intubation), management of SARS‐CoV‐2 co‐infection, and respiratory syncytial virus prevention.

**Aim:**

This article outlines the methodology used in the guideline update, following AGREE and CheckUp reporting standards.

**Methods:**

A Guideline Advisory Group and Guideline Development Committee, consisting of 29 clinical and methodological experts from Australasia, developed the guideline with consumer input. Forty‐one scoping questions on 25 topics were investigated. Systematic searches were performed by a subject librarian across five electronic databases (last search 24/01/24). Screening of 13,932 new articles occurred independently, in duplicate in two stages. The results of 431 articles were synthesized narratively and quantitatively where appropriate, per topic. Risk of bias was evaluated using appropriate tools. GRADE methodology was used to assess evidence quality and develop the recommendations, which were finalised through consensus voting across three guideline development meetings.

**Results:**

The update produced 41 recommendations (33 evidence‐based, 8 consensus‐based; including 11 new and 7 with key revisions), covering bronchiolitis investigations, management and RSV prevention. Interest‐holder groups consulted on the recommendations.

**Conclusion:**

The Australasian Bronchiolitis Guideline was updated and expanded according to AGREE and CheckUp reporting standards.

**Summary**

- The methodology used to update and expand the scope of the PREDICT Australasian bronchiolitis guideline is described with key steps including: (1) determining the guideline contributors; (2) defining the scope of the guideline update; (3) identifying, appraising, and synthesising the overall evidence; (4) revising or formulating new recommendations; and (5) interest‐holder consultation.
- We discuss strategies and considerations in performing a guideline update involving an expanded scope, a large literature review (approximately 14 000 articles), and development of 41 recommendations applicable to diverse paediatric care settings.
- This paper provides detailed guidance on how to update and increase the scope of a large clinical practice guideline, utilising the AGREE and CheckUp reporting standards. The Guideline is available online: https://www.predict.org.au/bronchiolitis‐guideline/.

## 1 Introduction

Bronchiolitis is the leading cause of hospital admission in infants in Australasia (Australia and Aotearoa New Zealand (AoNZ)) [1, 2]. It is a seasonal, acute respiratory condition triggered by a viral infection and characterised by the presence of diffuse crackles/wheeze on auscultation, and signs of a respiratory tract infection. Despite bronchiolitis being very common, its care has historically been affected by clinical practice variation, involving the use of ineffective or at times, harmful therapies [3, 4].

To address the variation in practice, the Paediatric Research in Emergency Departments International Collaborative (PREDICT) developed the first Australasian Bronchiolitis Guideline in 2016, which aimed to provide evidence‐based guidance on the management of infants (< 12 months of age) presenting to emergency departments (EDs) or hospitalised with bronchiolitis in Australia or AoNZ [5, 6].

In 2022, PREDICT initiated an update to the guideline to incorporate new evidence and to expand the scope to include intensive care (up to intubation), respiratory syncytial virus (RSV) vaccination, and SARS‐CoV‐2 co‐infection treatment. The expanded scope aimed to: (1) broaden the guideline's applicability to different settings and levels of care, recognising that escalation of care for severe illness occurs outside of the intensive care unit (ICU) setting, especially in regional centres; (2) address the changing landscape of managing acute respiratory infections in infants following the SARS‐CoV‐2 pandemic; and (3) acknowledge the roll‐out of maternal and infant RSV vaccines/immunotherapy in Australia. The target audience of the updated guideline includes clinicians working in EDs, paediatric wards, and ICUs who manage the care of infants with bronchiolitis in Australasian tertiary, regional, and rural hospitals.

This article describes the methodology and strategies used in performing the update of the expanded guideline covering approximately 14 000 articles over 25 topics, through to the development of 41 recommendations applicable to diverse care settings. A companion article reports an overview of the guideline recommendations [7]. The full guideline report and evidence profiles are available elsewhere [8, 9].

## 2 Methodology of the Guideline Update

The guideline update was prospectively registered on the Guideline International Network (GIN) library [10] and PROSPERO (CRD42023463917) [11]. The Appraisal of Guidelines Research and Evaluation (AGREE) and Checklist for the Reporting of Updated Guidelines (CheckUp) reporting standards were followed (Supporting Information S1: Appendix 1) [12, 13].

### 2.1 Determining the Guideline Contributors

The co‐leads of the guideline revision (MLB, SRD) established the Guideline Advisory Group (GAG) to oversee the update. It was comprised of five medical and two nursing specialists in general paediatrics and paediatric emergency medicine (MLB, FEB, EC, SRD, LH, SO, EO), and three guideline methodology experts (KL, EJT, CW). The GAG managed the evidence review, recommendation development, interest‐holder consultation, conflicts of interest, publication, and dissemination of the update. A consultative Guideline Development Committee (GDC) was formed to review the guideline scope and evidence, and to assist with finalising the recommendations. The GDC consisted of an additional 19 clinical and academic experts in paediatric emergency medicine, immunology, neonatology, intensive care, general paediatrics, and general practice from medical or nursing backgrounds. The GAG and GDC were gender balanced, with representation from Australia and AoNZ, tertiary and regional hospitals, and clinicians who provided Indigenous expertise. Further information about the guideline contributors is reported in Supporting Information S1: Appendix 2. Ten of 29 contributors (7 GAG, 3 GDC) were involved in developing the initial guideline. Contributors were assigned to one of four working groups based on their expertise (Supporting Information S1: Appendix 3).

Declarations of interest were collected using a standardised form that assessed for the presence of relationships and/or resourcing from entities that could be perceived to influence the guideline's output or to be financially affected by the guideline. The form was administered prior to appointment on the GAG or GDC and prior to each guideline development meeting. The information was used to judge the presence and severity of conflicts of interest (COIs) by the guideline co‐chairs (MLB, SRD), and a GAG member with extensive experience in managing COIs in clinical guideline development (CW). Multiple strategies protected against the influence of any COIs, including: (1) the disclosure of all COIs at the outset of a meeting; (2) individuals with moderate COIs abstaining from voting for the relevant topic, along with the deliberate inclusion of additional experts on that topic without COIs; (3) exclusion of individuals judged to have a significant COI from the GAG or GDC. We also used consensus voting procedures involving an 80% agreement threshold for approval of guideline topics, questions, outcomes, and recommendations. This method provided further protection from any COI that an individual might have. The primary systematic reviewer across all guideline topics (KL) did not have any conflicts of interest. All findings were thoroughly reviewed and deliberated by the GAG and GDC, where on balance there were minimal conflicts of interest (Supporting Information S1: Appendix 2, Tables A2–3), with most members having no conflicts to report. Only two GDC members were required to abstain from voting (topic: RSV vaccination); additional experts in the area without COIs were present. No COIs were of a severity to warrant exclusion from the GAG or GDC.

### 2.2 Defining the Scope of the Guideline Update

The scope of the guideline update was determined through consensus agreement of the GAG and GDC. The scope was broadened from the initial guideline to include intensive care (up to intubation), maternal and infant RSV vaccination/immunotherapy, and SARS‐CoV‐2 co‐infection treatment. The guideline co‐chairs developed a preliminary list of scoping questions either selected from the original guideline or developed for the update in population, intervention, comparator, outcome (PICO) format. The scoping questions were refined by the GAG, and finalised by the GDC through discussion and consensus voting (involving an 80% agreement threshold) in a virtual meeting. Thirty scoping questions were selected from the original guideline, and 11 new questions were developed to cover the expanded scope (Table 1). Three scoping questions from the initial guideline were excluded from the update, which covered home oxygen and beta2 agonist use in older infants (aged 12–24 months). Following consultation with medical colleges, societies, hospitals, and local governance groups, the scoping questions on beta2 agonist use in older infants were removed from the guideline scope. Overall, 41 scoping questions were investigated on 25 topics.

**TABLE 1:** Scoping questions for the guideline update.

Topic	No.	Question	Critical outcomes	Important outcomes
*Diagnosis*				
Physical examination and history	1.	In infants presenting to hospital, what factors in history and physical examination contribute to a differential diagnosis of bronchiolitis?	Diagnosis of bronchiolitis Sensitivity and specificity	—
Risk factors	2.	In infants presenting to hospital with bronchiolitis, what are the risk factors for admission or severe disease (e.g., prolonged length of hospital stay, ICU admission, and death)?	Admission to ICU Death Mechanical ventilation	Admission to hospital Length of stay Time on positive pressure ventilation support
CXR	3a.	In infants presenting to hospital or hospitalised with bronchiolitis, does performing a CXR, at the time of presentation or admission, beneficially change medical management or clinically relevant endpoints?	Diagnostic accuracy (3a–c) Indicator for administration of antibiotics (3a) Length of stay (3b,c) Length of ICU stay (3b,c)	Cost‐effectiveness (3a–c) Readmission (3a) Indicator for administration of antibiotics (3b,c)
	3b.	In infants who have an unexpected deterioration with bronchiolitis, does performing a CXR beneficially change medical management or clinically relevant endpoints?		
	3c.	In infants severely unwell with bronchiolitis (HDU/ICU level care), does performing a CXR beneficially change medical management or clinically relevant endpoints?		
Laboratory tests	4a.	In infants presenting to hospital or hospitalised with bronchiolitis, does performing laboratory tests (blood and/or urine), at the time of presentation or admission, beneficially change medical management or clinically relevant endpoints?	Length of stay (4a–c) Death (4a–c)	Length of ICU stay (4a–c) Diagnosis of bacterial co‐infection (including pneumonia and UTI) (4a–c)
	4b.	In infants who have an unexpected deterioration with bronchiolitis, does performing laboratory tests (blood and/or urine) beneficially change medical management or clinically relevant endpoints?		
	4c.	In infants severely unwell with bronchiolitis (HDU/ICU level care), does performing laboratory tests (blood and/or urine) beneficially change medical management or clinically relevant endpoints?		
Virological investigations	5.	In infants presenting to hospital or hospitalised with bronchiolitis, does performing virological investigations beneficially change medical management or clinically relevant endpoints?	Rate of hospitalisation Death Rate of ICU admission	Length of stay Length of ICU stay
*Management*				
Bronchiolitis scoring systems	6.	For infants presenting to hospital or hospitalised with bronchiolitis, does use of a bronchiolitis scoring system beneficially change medical management or clinically relevant endpoints?	—	Length of stay Diagnostic accuracy
Criteria for safe discharge	7.	For infants presenting to hospital or hospitalised with bronchiolitis, what criteria should be used for safe discharge?	Length of stay Readmission rate	—
Beta2 agonists	8a.	In infants presenting to hospital or hospitalised with bronchiolitis, does administration of beta2 agonists (nebulisation, aerosol, oral or IV) improve clinically relevant endpoints?	Rate of hospitalisation (8a,b) Length of stay (8a,b) Mechanical ventilation (8a,b)	Rate of readmission (8a,b) Adverse events (8a,b) Time on positive pressure ventilation support (8a,b)
	8b.	In infants presenting to hospital or hospitalised with bronchiolitis, with a personal or family history of atopy, does administration of beta2 agonists (nebulisation, aerosol, oral or IV) improve clinically relevant endpoints?		
Adrenaline/epinephrine	9.	In infants presenting to hospital or hospitalised with bronchiolitis, does administration of adrenaline/epinephrine (nebulisation, MDI, IM, or IV) improve clinically relevant endpoints?	Rate of hospitalisation Length of stay Mechanical ventilation	Rate of readmission Adverse events Time on positive pressure ventilation support
Hypertonic saline	10.	In infants presenting to hospital or hospitalised with bronchiolitis, does administration of nebulised hypertonic saline improve clinically relevant endpoints?	Rate of hospitalisation Length of stay Mechanical ventilation	Rate of readmission Adverse events Time on positive pressure ventilation support
Glucocorticoids	11a.	In infants presenting to hospital or hospitalised with bronchiolitis, does administration of systemic or local glucocorticoids (nebulisation, oral, IM or IV) improve clinically relevant endpoints?	Rate of hospitalisation (11a–c) Length of stay (11a–c) Mechanical ventilation (11a–c)	Rate of readmission (11a–c) Adverse events (11a–c) Time on positive pressure ventilation support (11a–c)
	11b.	In infants presenting to hospital or hospitalised with bronchiolitis, with a positive response to beta2 agonists, does administration of systemic or local glucocorticoids (nebulisation, oral, IM or IV) improve clinically relevant endpoints?		
	11c.	In infants presenting to hospital or hospitalised with bronchiolitis, does administration of the combination of systemic or local glucocorticoids (nebulisation, oral, IM or IV) and adrenaline improve clinically relevant endpoints?		
Supplemental oxygen and saturation targets	12a.	In infants presenting to hospital or hospitalised with bronchiolitis, does administration of supplemental oxygen improve clinically relevant endpoints?	Rate of hospitalisation (12a,b) Length of stay (12a,b)	Oxygen saturation target (12a,b) Feeding difficulties (12a,b) Readmission (12a,b)
	12b.	In infants presenting to hospital or hospitalised with bronchiolitis, what level of oxygen saturation should lead to commencement or discontinuation of supplemental oxygen to improve clinically relevant endpoints?		
Continuous pulse oximetry	13.	In infants hospitalised with bronchiolitis does continuous monitoring of pulse oximetry beneficially change medical management or clinically relevant endpoints?	Length of stay	Thresholds for discharge Frequency of nocturnal desaturations Maintenance of feeding
High flow therapy	14.	In infants hospitalised with bronchiolitis does the use of high flow nasal cannula improve clinically relevant endpoints?	Length of stay Rate of ICU admission Mechanical ventilation	Adverse events Cost‐effectiveness Time on positive pressure ventilation support
Chest physiotherapy	15.	In infants hospitalised with bronchiolitis, does chest physiotherapy improve clinically relevant endpoints?	Length of stay Length of oxygen therapy Mechanical ventilation	Adverse events Oxygen saturation levels Time on positive pressure ventilation support
Suctioning	16a.	In infants hospitalised with bronchiolitis, does suctioning of the nose or nasopharynx improve clinically relevant endpoints?	Length of stay (16a,b)	Adverse events (16a,b) Time on positive pressure ventilation support (16a,b)
	16b.	In infants hospitalised with bronchiolitis, does deep suctioning in comparison to superficial suctioning beneficially improve clinically relevant endpoints?		
Nasal saline	17.	In infants hospitalised with bronchiolitis, does the use of nasal saline drops improve clinically relevant endpoints?	Length of stay	Adverse events Oxygen saturation levels
CPAP	18.	In infants hospitalised with bronchiolitis, does the use of CPAP improve clinically relevant endpoints?	Length of stay Rate of ICU admission Mechanical ventilation	Adverse events Cost‐effectiveness Time on positive pressure ventilation support
Antibiotic medication	19a.	In infants presenting to hospital or hospitalised with bronchiolitis, does the use of antibiotic medication improve clinically relevant endpoints?	Length of stay (19a,b) Rate of ICU admission (19a,b) Mechanical ventilation (19a,b) Bronchiectasis (19c)	Adverse events (19a–c) Readmission within 6 months (19a–c) Persistent respiratory symptoms (19a–c) Time on positive pressure ventilation support (19a,b)
	19b.	In infants presenting to hospital or hospitalised with bronchiolitis, does the use azithromycin medication improve clinically relevant endpoints?		
	19c.	In infants presenting to hospital or hospitalised with bronchiolitis, does the use of antibiotic medication in infants who are at risk of developing bronchiectasis, improve clinically relevant endpoints?		
Non‐oral hydration	20a.	In infants presenting to hospital or hospitalised with bronchiolitis, does the use of non‐oral hydration improve clinically relevant endpoints?	Length of stay (20a–e) Mechanical ventilation (20a–e) Rate of ICU admission (20a,e)	Adverse events (20a,b,e) Rate of readmission (20b) Hyponatremia (20c,d) Time on positive pressure ventilation support (20a–e) Success of insertion (20b)
	20b.	In infants presenting to hospital or hospitalised with bronchiolitis, what forms of non‐oral hydration improve clinically relevant endpoints?		
	20c.	In infants presenting to hospital or hospitalised with bronchiolitis, does limiting the volume of non‐oral hydration impact on clinically relevant endpoints?		
	20d.	In infants presenting to hospital or hospitalised with bronchiolitis, does the type of IV fluid impact clinically relevant endpoints?		
	20e.	In infants presenting to hospital or hospitalised with bronchiolitis and managed with high flow therapy, does the use of enteral nutrition (oral and non‐oral) impact clinically relevant endpoints?		
Infection control practices	21.	In infants presenting to hospital or hospitalised with bronchiolitis, do infection control practises improve clinically relevant endpoints?	Nosocomial infection Length of stay	Adverse events Cost‐effectiveness
SARS‐CoV‐2 co‐infection and treatment	22a.	In infants presenting to hospital or hospitalised with bronchiolitis, to what extent does SARS‐CoV‐2 virus infection, or co‐infection, contribute to disease incidence or severity?	Length of stay (Q22a,b) Rate of ICU admission (Q22a) Rate of hospitalisation (Q22b) Mechanical ventilation (Q22b)	Adverse events (Q22a,b) Rate of readmission (Q22b) Time on positive pressure ventilation support (Q22b)
	22b.	In infants presenting to hospital or hospitalised with bronchiolitis, who test positive for SARS‐CoV‐2, does use of therapies targeting the SAR‐CoV‐2 virus (steroids, antivirals) improve clinically relevant endpoints?		
*RSV prevention*				
Monoclonal antibody therapy	23.	Which infants at risk of serious outcomes from bronchiolitis, have clinically relevant benefit from monoclonal antibody therapy (e.g., palivizumab)?	Admission to hospital with bronchiolitis Admission to ICU with bronchiolitis Mechanical ventilation	Adverse events (infant) Medical visit (ED/acute care/GP) for bronchiolitis Cost‐effectiveness Time on positive pressure ventilation support
Maternal RSV immunisation	24.	Does universal maternal antenatal RSV immunisation result in clinically relevant benefit for infants?	Admission to hospital with bronchiolitis Admission to ICU with bronchiolitis Mechanical ventilation	Adverse events (maternal/infant) Medical visit (ED/acute care/GP) for bronchiolitis Cost‐effectiveness Time on positive pressure ventilation support
Infant RSV immunisation	25.	Does universal infant RSV immunisation result in clinically relevant benefit?	Admission to hospital with bronchiolitis Admission to ICU with bronchiolitis Mechanical ventilation	Adverse events (infant) Medical visit (ED/acute care/GP) for bronchiolitis Cost‐effectiveness Time on positive pressure ventilation support

*Note:* The patient group was infants aged < 12 months with bronchiolitis, apart from questions 23 (all infants), 24 (pregnant women), and 25 (all infants).

Abbreviations: CPAP = Continuous positive airway pressure; CXR = Chest x‐ray; ED = Emergency department; GP = General practice; HDU = High dependency unit; ICU = Intensive care unit; IM = Intramuscular; IV = Intravenous; MDI = Metered dose inhaler; RSV = Respiratory syncytial virus; SARS‐CoV‐2 = Severe acute respiratory syndrome coronavirus 2; UTI = Urinary tract infection.

The critical and important outcomes for each scoping question were determined a priori by the GAG and GDC. A preliminary list of outcomes was generated for each question from the initial guideline and key research articles on the new topics by the guideline co‐chairs. These outcomes were independently reviewed, and subsequently voted on and prioritised by the GAG and GDC in a virtual meeting where an 80% agreement threshold was required. This process resulted in a list of outcomes and their priority per topic. The importance of these outcomes was subsequently confirmed in Australasian families with recent bronchiolitis experience through qualitative interviews (Supporting Information S1: Appendix 2) [14].

### 2.3 Identifying, Appraising, and Synthesising the Overall Evidence

Systematic searches were performed by a subject librarian using five electronic databases: Ovid MEDLINE, Ovid EMBASE, PubMed, CINAHL, and the Cochrane Library (last search 24/01/24) (Supporting Information S1: Appendix 4). The systematic search strategy was adapted to include new terms to support the expanded guideline scope. We conducted one large systematic search covering all 25 topics for each database, with the results limited to the English language and publication date (year 2000 to present). Supplementary searches were run for new topics (ICU care, RSV monoclonal antibodies/vaccination) that were backdated to the search period of the initial guideline (2010–2014). The included articles of the 2016 guideline were screened for evidence on ICU outcomes. Manual searches were performed from trial registrations and conference abstracts, and the reference lists of recent bronchiolitis guidelines and systematic reviews. Subject matter experts within the GAG/GDC reviewed the final list of included articles to ensure no key studies were missing.

Study selection occurred in two stages involving title and abstract, and full text screening using Covidence software (Veritas Health Innovation, Melbourne, Australia) [15]. The screening was performed independently in duplicate by two of 10 reviewers against unique eligibility criteria per topic. During the full‐text screen, nine of 10 reviewers (MLB, FEB, EC, SRD, LH, SO, EO, EJT, CW) screened the articles tagged to their topic groups in Covidence, while one reviewer (KL) screened across all topics. Training was held prior to each screening stage, and inter‐rater agreement was monitored. Conflicts were resolved by a third reviewer not involved in the initial dispute (MLB, SRD, KL, EJT).

Eligible systematic reviews and primary studies were included for each topic. Where a recent, comprehensive systematic review existed for a topic, this was extracted and used to inform the recommendation. Eligible primary studies not covered in the review were further extracted. In the absence of an eligible, recent, comprehensive systematic review, the research team performed their own synthesis of the primary studies. The result of this method was that all eligible primary studies for a topic were included and synthesised to inform the recommendations. We implemented multiple strategies to avoid introducing bias from double‐counting outcome data, as per Cochrane guidance [16]. These included: (1) selecting the most high‐quality, comprehensive, and recent systematic review for extraction where there were multiple eligible reviews reporting on overlapping evidence; (2) extracting from eligible, non‐overlapping systematic reviews.

Data extraction and risk of bias appraisals were performed by one reviewer (KL), with review by a topic expert (MLB, FEB, EC, SRD, LH, SO, EJT, CW). The data were extracted into standardised spreadsheet forms. The risk of bias of the included studies was assessed using tools appropriate to the evidence. The tools included the Cochrane Risk of Bias 2 (RoB2) [17], Risk Of Bias In Non‐randomised Studies of Interventions (ROBINS‐I) [18], Risk Of Bias In Non‐randomised Studies—of Exposures (ROBINS‐E) [19], RoB2 tool for crossover trials [20], Prediction model Risk Of Bias ASessment Tool (PROBAST) [21], Joanna Briggs Institute Checklist for Prevalence Studies [22], and the Newcastle Ottawa Quality Assessment Scale for Cohort Studies [23]. Where systematic reviews were included, the risk of bias ratings that the review reported were extracted. Cost‐effectiveness studies were not evaluated for risk of bias per Cochrane guidance [24].

The data were synthesised narratively and quantitatively through meta‐analysis where appropriate, using Review Manager Web software (RevMan Web) (The Cochrane Collaboration, London, United Kingdom), and following Cochrane criteria for appropriateness [25]. The data were quantitatively synthesised using fixed‐effect or random‐effects meta‐analyses, depending on the presence of clinical or methodological heterogeneity. A fixed‐effect model was chosen where it could be assumed that the trials were estimating the same underlying treatment effect. A random‐effects model was used where heterogeneity was sufficient to expect that the treatment effects were related but different between trials. Statistical heterogeneity was assessed visually through forest plots and using the I^2^ statistic. We expressed dichotomous outcomes as risk ratios (RR) or odds ratios (OR), and continuous outcomes as mean difference (MD) with 95% confidence intervals. Subgroup and sensitivity analyses were performed where there were sufficient data to meaningfully explore possible sources of heterogeneity, and to assess the robustness of results (e.g., through excluding studies at high risk of bias, estimated means).

The quality and certainty of the body of evidence was evaluated using GRADE methodology with GRADEpro GDT software (McMaster University and Evidence Prime Inc., Hamilton, ON, Canada) [26, 27]. The quality of the evidence for each outcome within a topic was determined through downgrading for risk of bias, inconsistency, indirectness, imprecision, and publication bias, to result in an overall rating of high, moderate, low, or very low quality. Evidence from non‐randomised studies started at low quality for topics that could include RCT evidence. If there were no downgrades, this evidence could be upgraded based on GRADE criteria (large magnitude of effect, dose–response gradient, plausible confounding). The appraisals were performed by one reviewer (KL), with each appraisal reviewed by a topic expert (MLB, FEB, EC, SRD, LH, SO, EJT, CW). Evidence tables were generated for each comparison within a topic. An overall evidence quality rating was determined for each topic from the lowest rating of its critical outcomes, following GRADE guidance [26]. The overall evidence quality ratings for a topic could range from high to very low.

### 2.4 Revising or Formulating New Recommendations

The GRADE evidence‐to‐recommendation framework was used to develop the recommendations and determine the recommendation strength. The framework required the GAG/GDC to consider the evidence quality, balance of benefits and harms, resource implications, feasibility in the Australasian context, acceptability, end‐user values and preferences, and equity and human rights implications of the proposed action. The judgements for each domain were presented in an evidence‐to‐recommendation table per recommendation. These judgements informed the recommendation strength (Table 2), which determined the language of the recommendation (e.g., “do not,” or “use” for strong recommendations, “do not routinely” or “consider” for conditional or weak recommendations). In the absence of evidence, consensus‐based recommendations were developed. The overall evidence and draft recommendations were presented in an evidence profile per topic.

**TABLE 2:** Recommendation strength definitions.

Recommendation strength	Definition
Strong 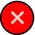 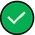	The GDC is confident that the desirable effects of the action outweigh its undesirable effects, or vice versa. Most or all individuals will be best served by the recommended course of action.
Weak 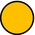	The desirable effects of the action probably outweigh the undesirable effects, or vice versa, but appreciable uncertainty exists. Not all individuals will be best served by the recommended course of action.
Conditional 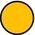	A *weak* recommendation where the recommended course of action may depend on patient factors, resources or setting.
Consensus‐based recommendation 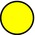	A recommendation formulated through GDC consensus in the absence of evidence, where a systematic review of the evidence was conducted as part of the search strategy.

Abbreviation: GDC, Guideline Development Committee.

The recommendations were finalised through discussion and consensus voting across three guideline development meetings (two virtual, one in‐person), with the GAG/GDC reviewing the evidence profiles prior to each meeting. During the meetings, the evidence for each topic was presented with discussion on the components of the evidence‐to‐recommendation table and the recommendation wording, before voting to finalise the recommendation. A threshold of 80% agreement was required for a recommendation to pass. If the agreement threshold was not reached, the recommendation was revised by the GAG and re‐voted on at the following meeting. Four of 25 topics required more than one round of voting (CXR, discharge criteria, glucocorticoids, nasal saline), due to changes required to the recommendation wording. Consensus was reached on all topics.

This process resulted in the development of 11 new recommendations, key changes to seven recommendations, and 25 recommendations that were unchanged or underwent minor revisions from the initial guideline (detailed elsewhere [7, 8]).

### 2.5 Interest‐Holder Consultation

Interest‐holder groups provided consultation to ensure the acceptability, feasibility, and applicability of the recommendations to Australasian consumers of the guideline (clinicians, policymakers, patients and families). Ten colleges, five societies, and 13 hospitals/local governance groups were invited to provide consultation by email letter over an 8‐week period, with feedback received from six colleges, four societies, and ten hospitals/local governance groups. For four hospitals, more than one unit provided consultation. A preliminary version of the report and bedside summary document was circulated to interest‐holders for review. Feedback was sought on errors of fact, clarifications, and considerations on the conditions in which the recommendations apply or how they are implemented. The feedback from the consultation was tabulated in a spreadsheet and integrated into the report. The interest‐holder groups were provided with a letter documenting the response to their feedback and a copy of the final guideline.

In‐depth qualitative interviews were conducted with families with a recent hospital experience of bronchiolitis in Australia or AoNZ, to understand values and preferences for bronchiolitis care (Supporting Information S1: Appendix 2) [14]. A purposive sampling approach was taken to ensure equal representation from Indigenous (Māori, Pasifika, Aboriginal/Torres Strait Islander) and non‐Indigenous families from Australia and AoNZ, who were discharged from ED or admitted to hospital in a metropolitan or regional hospital. The interviews helped to confirm the importance of the guideline outcomes and recommendation strength, and provided insights to support guideline implementation. Further to this, a patient family group is advising on the implementation of the guideline in regional and rural settings (via the Regional and Rural Translation‐Bronchiolitis (RART‐Bronch) study).

## 3 Strategies and Considerations

### 3.1 Retrieving and Screening a Large Volume of Evidence (Approximately 14 000 Records) on 25 Topics Within a Short Timeframe

We adopted several strategies to efficiently search and screen a large volume of evidence across 25 topics (41 PICO questions). Our evidence retrieval involved a large systematic search covering all topics, as opposed to 25 separate searches. This strategy was chosen to avoid unnecessary duplication of screening across topics, and was practical with one team screening the evidence.

The 13 932 search results were screened in duplicate by 10 reviewers from the GAG. All reviewers assessed the records for eligibility across the 25 topics during the title and abstract screen. To reduce error that could be introduced from screening many topics with different eligibility criteria simultaneously, the reviewers used a reference guide summarising the eligibility criteria per topic. During the full‐text screen, reviewers screened the full‐text articles tagged to their working groups.

### 3.2 Methodological Changes for the Guideline Update

The guideline update included several methodological changes. New software, risk of bias tools, and reporting requirements were used, most of which were released subsequent to the initial guideline [12, 13, 15, 17, 18, 19, 20, 21, 27]. As the evidence had matured, we were able to perform quantitative syntheses for several topics. We introduced evidence profiles that synthesised the initial and new evidence per topic in detail for decision‐makers. The evidence profiles were published in the guideline report appendix as a detailed reference for guideline consumers.

### 3.3 Developing Recommendations Applicable to Varied Settings and Consumers

The guideline scope required producing recommendations applicable to different clinicians (medical and nursing), hospital units (ED, ward, ICU), and tertiary and regional hospitals with varied access to types of care (e.g., ICU). The recommendations also needed to be applicable to Australia and AoNZ, where there was variation in the public funding of interventions (e.g., RSV vaccinations). To address this, scoping questions were introduced that focused on the evidence for specific settings (e.g., ICU care). We also ensured representation of diverse perspectives on the GAG/GDC and in the interest‐holder groups approached for consultation. The GRADE evidence‐to‐recommendation framework required consideration of health equity and accessibility challenges associated with a recommendation during its development. Guideline group remarks on considerations for applying the recommendations in varied settings or subgroups were presented alongside the recommendations in the report and were documented in each evidence profile.

### 3.4 Developing Recommendations Around Inconsistent Illness Severity Definitions in the Literature

Our evidence evaluation was complicated by inconsistent definitions of bronchiolitis illness severity in the literature. To address this, we developed a table to define illness severity levels based on clinical criteria reported within the paediatric early warning score tools used in Australasian hospitals and the eligibility criteria of included studies. The definitions were reviewed by the GAG/GDC and interest‐holder groups. The definitions applied to all recommendations that specified actions for infants with bronchiolitis of a particular severity. Illness severity could not be simplified to a level of care received (e.g., ICU care), as the recommendations needed to apply to regional hospitals where patients may not have immediate ICU access.

### 3.5 Developing Recommendations in the Context of Evolving Evidence

Several recommendations needed to be developed in the context of evolving evidence. Some topics appeared to regularly have new evidence available (e.g., RSV vaccination), while other topics had ongoing studies that could change the strength of the recommendations in the near future (e.g., combined glucocorticoid and adrenaline/epinephrine therapy). Although recommendations were made based on the current evidence, the GAG determined that several of these topics should be adapted to a “living guideline” format in the near future. A living guideline format would allow for regular updates to the recommendations as new evidence becomes available that may change the recommendation, or improve the certainty of evidence.

## 4 Strengths and Limitations

Our methodology enabled us to develop a large number of recommendations (41) applicable to diverse hospital settings (e.g., metropolitan, regional), units (e.g., ED, ward, ICU), and patients (e.g., demographics, underlying health conditions), within a two‐year time period. The resourcing to achieve this involved: (1) one full‐time research fellow experienced in guideline development and evidence synthesis; (2) financing for an in‐person guideline development meeting, including travel and accommodation for committee members from Australia and AoNZ; (3) financing and labour to recruit, conduct, and analyse 25 30–45 min qualitative interviews in families; (4) software licences (e.g., Covidence, RevMan); (5) institutional access to a subject librarian and electronic database results; (6) a web developer to create the guideline website. This was supplemented by labour from the GAG and GDC that was either funded indirectly through other sources or voluntary.

Although our guideline committee included clinicians who work extensively with migrant communities, and are located in regions with a high proportion of migrant families (e.g., Auckland, Melbourne), we did not record the migrant status of the committee members, and therefore, are unable to comment on how migrant perspectives may have influenced the recommendations. However, our consultation with patient families included an equal proportion of Pasifika families (due to their increased risk of severe bronchiolitis) with a migrant background, who contributed to our understanding of family values and preferences for bronchiolitis care in hospital. Our consultation did not specifically address other migrant families, although these families do not have the same risk as indigenious and Pasifika families. A further limitation of our approach is that there are other types of interest‐holders that we could have included in the development of our guideline, such as policymakers and healthcare payers. Instead, publicly available, published information was relied upon to inform the committee's discussions of relevant policy, approvals, and costs of considered interventions.

## 5 Conclusion

This article covers the methodology used in updating the expanded PREDICT Australasian Bronchiolitis Guideline in 2025. This detailed guidance may be of value to those updating and expanding the scope of a large clinical practice guideline applicable to diverse care settings.

## Conflicts of Interest

M.L.B., S.R.D., F.E.B., S.O., and E.O.’s institutions have received equipment from Fisher and Paykel Healthcare to support bronchiolitis research. S.R.D. has received funding from Fisher and Paykel Healthcare for travel to an international meeting discussing high flow therapy. None of the authors receive any personal financial benefits from industry sponsors.

## Supporting information


**Data S1.** Supporting Information.

## Data Availability

Data extraction forms can be accessed through a request to the corresponding author.
