# Supplementary material for: Methodology of the Updated and Expanded Australasian Bronchiolitis Guideline
Source: J Paediatr Child Health. 2025 Jul 17;61(8):1169–81. doi: 10.1111/jpc.70145 (PMC12397842; doi:10.1111/jpc.70145)
Supplement: Supplementary file 1 — Data S1. Supporting Information. [file JPC-61-1169-s001.docx]

Loveys K., Tavender E.J., Babl F.E., Cotterell E., Haskell L., O’Brien S., Oakley E., Wilson C., Borland, M.L., Dalziel, S.R., on behalf of the PREDICT Network. Methodology of the updated and expanded Australasian Bronchiolitis Guideline. Journal of Paediatrics and Child Health (2025): 1-12. Doi:10.1111/jpc.70145.

# Supplementary Material

**Contents**

[Appendix 1. CheckUp checklist 2](#_Toc184891552)

[Appendix 2. Guideline contributors and declarations of interest 3](#_Toc184891553)

[Appendix 3. Guideline topics by working group 7](#_Toc184891554)

[Appendix 4. Systematic search strategies 8](#_Toc184891555)

## Appendix 1. CheckUp checklist

*Table A1. Completed checklist for the reporting of updated guidelines (CheckUp)*

| ITEM NO. | ITEM | ASSESSMENT | REPORTED ON PAGE NUMBER | NOTES |
| --- | --- | --- | --- | --- |
| 1 | The updated version can be distinguished from the previous version of the clinical guideline. | Yes  No  Unclear  Not applicable | Title, abstract (page 3),  introduction (page 5, paragraphs 2, 3) |  |
| 2 | The rationale for updating the clinical guideline is reported. | Yes  No  Unclear  Not applicable | Abstract (page 3),  introduction (page 5, paragraph 3) |  |
| 3 | Changes in the scope and purpose between the updated and previous version are described and justified. | Yes  No  Unclear  Not applicable | Introduction (page 5, paragraph 3); Section 2.2 Defining the scope of the guideline update (page 6) |  |
| 4 | The sections reviewed in the updating process are described. | Yes  No  Unclear  Not applicable | Section 2.1 Defining the scope of the guideline update (page 6); Appendix 3, Table A4 Guideline Topics |  |
| 5 | Recommendations are clearly presented and labelled as new, modified, or not changed. Deleted recommendations are clearly noted. | Yes  No  Unclear  Not applicable | See notes | This information is presented in detail in a separate, complimentary article describing the recommendations from the guideline update, and the guideline main report:  Borland ML, Loveys K, Babl FE, Cotterell E, Haskell L, O’Brien S, et al. Australasian Bronchiolitis Guideline: 2025 Update. *Journal of Paediatrics and Child Health* (2025).  PREDICT network. Australasian Bronchiolitis Guideline: 2025 Update (PREDICT Network, 2025). <https://www.predict.org.au/wp-content/uploads/2025/02/ABG2025-Report-final.pdf> |
| 6 | Changes in recommendations are reported and justified. | Yes  No  Unclear  Not applicable | See notes | This information is presented in detail in a separate, complimentary article describing the recommendations from the guideline update, and the guideline main report:  Borland ML, Loveys K, Babl FE, Cotterell E, Haskell L, O’Brien S, et al. Australasian Bronchiolitis Guideline: 2025 Update. *Journal of Paediatrics and Child Health* (2025).  PREDICT network. Australasian Bronchiolitis Guideline: 2025 Update (PREDICT Network, 2025). <https://www.predict.org.au/wp-content/uploads/2025/02/ABG2025-Report-final.pdf> |
| 7 | The panel participants in the updated version are described. | Yes  No  Unclear  Not applicable | Supplementary Material, Appendix 2, Tables A2 and A3; Section 2.1 Determining the guideline contributors (pages 5, 6) |  |
| 8 | Disclosures of interests of the group responsible for the updated version are recorded. | Yes  No  Unclear  Not applicable | Section 2.1 Determining the guideline contributors (page 6); Supplementary Material, Appendix 2, Tables A2 and A3 |  |
| 9 | The role of the funding body for the updated version is identified and described. | Yes  No  Unclear  Not applicable | Funding section (page 12) |  |
| 10 | The methods used for searching and identifying new evidence in the updating process are described. | Yes  No  Unclear  Not applicable | Section 2.3 Identifying, appraising, and synthesizing the overall evidence (page 6); Supplementary Material, Appendix 4, Systematic search strategies |  |
| 11 | The methods used for evidence selection in the updating process are described. | Yes  No  Unclear  Not applicable | Section 2.3 Identifying, appraising, and synthesizing the overall evidence (page 6); See notes | Further detail of the specific eligibility criteria per topic is outlined in the PROSPERO registration (CRD42023463917). |
| 12 | The methods used to assess the quality of the included evidence in the updating process are described. | Yes  No  Unclear  Not applicable | Section 2.3 Identifying, appraising, and synthesizing the overall evidence (pages 7, 8) |  |
| 13 | The methods used for the evidence synthesis in the updating process are described. | Yes  No  Unclear  Not applicable | Section 2.3 Identifying, appraising, and synthesizing the overall evidence (pages 7, 8, 10) |  |
| 14 | The methods used for externally reviewing the updated version are described. | Yes  No  Unclear  Not applicable | Section 2.5 Interest-holder consultation (page 9) |  |
| 15 | The methods and plan for implementing the changes of the updated version in practice are described. | Yes  No  Unclear  Not applicable | See notes | This information is presented in detail in the guideline main report:  PREDICT network. Australasian Bronchiolitis Guideline: 2025 Update (PREDICT Network, 2025). <https://www.predict.org.au/wp-content/uploads/2025/02/ABG2025-Report-final.pdf> |
| 16 | The plan and methods for updating the new version in the future are reported. | Yes  No  Unclear  Not applicable | Section 3.5 Developing recommendations in the context of evolving evidence; See notes | This information is presented in detail in the guideline main report:  PREDICT network. Australasian Bronchiolitis Guideline: 2025 Update (PREDICT Network, 2025). <https://www.predict.org.au/wp-content/uploads/2025/02/ABG2025-Report-final.pdf> |

## Appendix 2. Guideline contributors and declarations of interest

Table A2. Guideline Advisory Group

| NAME | EXPERTISE | INSTITUTION | LOCATION | ROLE | INTERESTS DECLARED | COI CONCLUSION | DEVELOPED 2016 GUIDELINE |
| --- | --- | --- | --- | --- | --- | --- | --- |
| Meredith L Borland (Co-chair) | Paediatric emergency specialist | Perth Children’s Hospital; University of Western Australia | Perth, WA, Australia | Guideline co-chair, co-lead of topic group one | Meredith Borland’s institution has received equipment from Fisher and Paykel Healthcare to support bronchiolitis research. | Conflict of interest  No action required | Yes  No |
| Stuart R Dalziel (Co-chair) | Paediatric emergency specialist | Starship Children’s Hospital; The University of Auckland | Auckland, Aotearoa New Zealand | Guideline co-chair, co-lead of topic group four | Stuart Dalziel’s institution has received equipment from Fisher and Paykel Healthcare to support bronchiolitis research. Stuart Dalziel has received funding from Fisher and Paykel Healthcare for travel to an international meeting discussing HF therapy. | Conflict of interest  No action required | Yes  No |
| Franz E Babl | Paediatric emergency specialist | Royal Children’s Hospital; Murdoch Children’s Research Institute; The University of Melbourne | Melbourne, VIC, Australia | Co-lead of topic group three | Franz Babl’s institution has received equipment from Fisher and Paykel Healthcare to support bronchiolitis research. | Conflict of interest  No action required | Yes  No |
| Elizabeth Cotterell | Regional paediatrician | Armidale Rural Referral Hospital; The University of New England | Armidale, NSW, Australia | Co-lead of topic group two | None declared | No conflict of interest | Yes  No |
| Libby Haskell | Paediatric emergency nurse practitioner | Starship Children’s Hospital; The University of Auckland | Auckland, Aotearoa New Zealand | Co-lead of topic group two | None declared | No conflict of interest | Yes  No |
| Kate Loveys | Guideline methodology, evidence synthesis | The University of Auckland | Auckland, Aotearoa New Zealand | Primary reviewer across topics | None declared | No conflict of interest | Yes  No |
| Ed Oakley | Paediatric emergency specialist | Royal Children’s Hospital | Melbourne, VIC, Australia | Co-lead of topic group one | Ed Oakley’s institution has received equipment from Fisher and Paykel Healthcare to support bronchiolitis research. | Conflict of interest  No action required | Yes  No |
| Sharon O’Brien | Paediatric emergency nursing | Perth Children’s Hospital | Perth, WA, Australia | Co-lead of topic group four | Sharon O’Brien’s institution has received equipment from Fisher and Paykel Healthcare to support bronchiolitis research. | Conflict of interest  No action required | Yes  No |
| Emma J Tavender | Implementation science | Murdoch Children’s Research Institute; The University of Melbourne | Melbourne, VIC, Australia | Co-lead of topic group three | None declared | No conflict of interest | Yes  No |
| Catherine Wilson | Research coordination | Murdoch Children’s Research Institute | Melbourne, VIC, Australia | Co-lead of topic group three | None declared | No conflict of interest | Yes  No |

Table A3. Guideline Development Committee

| NAME | EXPERTISE | INSTITUTION | LOCATION | ROLE | INTERESTS DECLARED | COI CONCLUSION | DEVELOPED 2016 GUIDELINE |
| --- | --- | --- | --- | --- | --- | --- | --- |
| Jane Alsweiler | Neonatology | Starship Children’s Hospital; The University of Auckland | Auckland, Aotearoa New Zealand | Topic group four | Jane Alsweiler’s institution has received funding for RSV monoclonal antibodies research in infants and high risk children. | Conflict of interest  No action required | Yes  No |
| David Armstrong | Paediatric respiratory specialist | Monash Children’s Hospital | VIC, Australia | Topic group three | None declared | No conflict of interest | Yes  No |
| Simon Craig | Paediatric emergency specialist | Monash Medical Centre; Monash University | VIC, Australia | Topic group three | None declared | No conflict of interest | Yes  No |
| Nigel Crawford | Paediatric immunology specialist | Royal Children’s Hospital; Murdoch Children’s Research Institute; The University of Melbourne | VIC, Australia | Topic group four | None declared | No conflict of interest | Yes  No |
| Dianne Crellin | Paediatric emergency nurse practitioner | Royal Children’s Hospital; Murdoch Children’s Research Institute | VIC, Australia | Topic group four | None declared | No conflict of interest | Yes  No |
| Sonja Crone | Regional general paediatrician | Rotorua Lakes Hospital | Rotorua, Aotearoa New Zealand | Topic group two | None declared | No conflict of interest | Yes  No |
| Trevor Duke | Paediatric intensive care specialist | Royal Children’s Hospital; The University of Melbourne | VIC, Australia | Topic group three | None declared | No conflict of interest | Yes  No |
| Shane George | Paediatric emergency medicine and intensive care specialist | Gold Coast University Hospital | QLD, Australia | Topic group two | Shane George’s institution has received:   - Equipment and funding from Fisher and Paykel Healthcare to support bronchiolitis research. - Funding for EDP-938 phase 2 study (N-protein modulator) research - Funding for AK0529 (fusion inhibitor) phase 2 study research | Conflict of interest  Participation restricted (abstained from voting on relevant recommendations) | Yes  No |
| Christine Jeffries-Stokes | Regional general paediatrician | Kalgoorlie Hospital; The University of Western Australia | WA, Australia | Topic group one | None declared | No conflict of interest | Yes  No |
| Nidhi Krishnan | Paediatric emergency medicine trainee | Queensland Children’s Hospital | QLD, Australia | Topic group two | None declared | No conflict of interest | Yes  No |
| Anna Lithgow | General paediatrician | Royal Darwin Hospital | NT, Australia | Topic group two | None declared | No conflict of interest | Yes  No |
| Ken Peacock | Tertiary general paediatrician | Sydney Children’s Hospitals Network | NSW, Australia | Topic group one | None declared | No conflict of interest | Yes  No |
| Tomas Ratoni | Paediatric network clinical nurse consultant | Northern NSW Local Health District | NSW, Australia | Topic group two | None declared | No conflict of interest | Yes  No |
| Peter Richmond | General paediatrician and immunologist | Perth Children’s Hospital; The University of Western Australia | WA, Australia | Topic group four | Peter Richmond’s institution has received funding for:   - RSV maternal and paediatric vaccination research; - RSV monoclonal antibody research in infants and high risk children; - Development of a live commensal bacteria for the prevention of otitis media and viral respiratory infections;   Virtual lectures to RSV investigators in Merck monoclonal antibody trial (Merck) and Canadian health care workers on RSV prevention (Astra Zeneca). | Conflict of interest  Participation restricted (abstained from voting on relevant recommendations) | Yes  No |
| Annie Smith | Paediatric respiratory nurse | Southland Hospital | Invercargill, Aotearoa New Zealand | Topic group three | None declared | No conflict of interest | Yes  No |
| Rebecca Starkie | General practitioner | The University of Melbourne | VIC, Australia | Topic group four | None declared | No conflict of interest | Yes  No |
| David Thomas | Tertiary general paediatrician | Women’s and Children’s Hospital | SA, Australia | Topic group three | None declared | No conflict of interest | Yes  No |
| Alexandra Wallace | Regional general paediatrician | Waikato Hospital | Hamilton, Aotearoa New Zealand | Topic group one | None declared | No conflict of interest | Yes  No |
| Michael Zhang | Paediatric emergency specialist | John Hunter Hospital | NSW, Australia | Topic group one | None declared | No conflict of interest | Yes  No |

RSV = Respiratory syncytial virus

**Characteristics of the Guideline Advisory Group (GAG) and Guideline Development Committee (GDC): Supplementary Information**

- Indigenous representation: The GAG and GDC included clinicians and/or academics of Indigenous ethnicity, and clinicians who work extensively with Indigenous communities and are located in regions with a high proportion of Indigenous families (e.g., Darwin, Kalgoorlie, Auckland, Rotorua, Waikato).

**Patient consultation**

Patient consultation on the guideline occurred through in-depth qualitative interviews in families with a recent hospital experience of bronchiolitis in Australia or Aotearoa New Zealand (13). A purposive sampling approach was taken to ensure equal representation from Indigenous (Māori, Pasifika, Aboriginal/Torres Strait Islander) and non-Indigenous families in Australia or Aotearoa New Zealand who received care from regional or metropolitan hospitals, and whose infant was discharged from the emergency department or admitted to hospital. This approach enabled a diverse perspective. This was viewed as a more appropriate and accessible method for families to meaningfully engage in the guideline’s development, given the nature of bronchiolitis (an acute respiratory condition caused by viral infection, that families tend to have a brief experience with, e.g., one to two weeks), and the large size of the guideline requiring a substantial workload that may be impractical for families of infants (<12 months of age). The interviews helped to confirm the importance of the outcomes and the strength of the recommendations, and provided insights to support guideline implementation.

## Appendix 3. Guideline topics by working group

Table A4. Guideline topics

| Group one | Group two | Group three | Group four |
| --- | --- | --- | --- |
| Physical examination and history (R1) | Beta2 agonists (R8a-b) | Hypertonic saline (R10) | Non-oral hydration (R20a-e) |
| Risk factors (R2) | Adrenaline/epinephrine (R9) | Supplementary oxygen and saturation targets (R12a-b) | Infection control practices (R21) |
| CXR (R3a-c) | Glucocorticoids (R11a-c) | Continuous pulse oximetry (R13) | SARS-CoV-2 co-infection and treatment (R22a-b) |
| Laboratory tests (R4a-c) | Antibiotic medication (R19a,c) | High flow therapy (R14) | Infant RSV monoclonal antibody therapy (R23) |
| Virological investigations (R5) | Azithromycin (R19b) | Chest physiotherapy (R15) | Maternal active RSV immunisation (R24) |
| Bronchiolitis scoring systems (R6) | - | Suctioning (R16a-b) | Infant active RSV immunisation (R25) |
| Criteria for safe discharge (R7) | - | Nasal saline (R17) | - |
| - | - | Continuous positive airway pressure (R18) | - |

CXR = Chest x-ray; ED = Emergency department; RSV = Respiratory syncytial virus; SARS-CoV-2 = Severe acute respiratory syndrome coronavirus 2.

## Appendix 4. Systematic search strategies

**Ovid MEDLINE(R) ALL <1946 to June 19, 2023>**

Search date: 21/06/23

1 bronchiolitis/ or bronchiolitis, viral/ or respiratory syncytial viruses/ or respiratory syncytial virus, human/ or Respiratory Syncytial Virus Infections/ or (bronchiolit* or wheez* or (Respiratory adj1 Syncytial adj1 Virus*)).af. or rsv.tw. 48765

2 limit 1 to (case reports or comment or editorial or letter) 6402

3 limit 1 to (clinical trial, all or clinical trial, phase i or clinical trial, phase ii or clinical trial, phase iii or clinical trial, phase iv or clinical trial or controlled clinical trial or guideline or meta analysis or practice guideline or randomized controlled trial or "review" or systematic reviews) 8638

4 1 and exp Evidence-Based Medicine/ 135

5 (1 not 2) or 3 or 4 42783

6 *bronchiolitis, viral/bl, ci, cl, co, dg, di, dt, ep, et, ge, hi, im, mi, mo, pa, pp, pc, th, ur, vi 1012

7 *bronchiolitis/bl, ci, cl, co, dg, di, dt, ep, et, ge, hi, im, mi, mo, pa, pp, pc, th, ur, vi 2446

8 6 or 7 3450

9 limit 8 to (case reports or comment or editorial or letter) 805

10 limit 8 to (clinical trial, all or clinical trial, phase i or clinical trial, phase ii or clinical trial, phase iii or clinical trial, phase iv or clinical trial or controlled clinical trial or guideline or meta analysis or practice guideline or randomized controlled trial or "review" or systematic reviews) 877

11 8 and exp Evidence-Based Medicine/ 64

12 (8 not 9) or 10 or 11 2702

13 Natural History/ or exp Epidemiology/ 29277

14 exp "reproducibility of results"/ or (scoring adj1 system*).tw. 503239

15 "severity of illness index"/ or (disease adj1 severity).tw. 311661

16 diagnosis, differential/ 467890

17 physical examination/ or exp auscultation/ or blood pressure determination/ or exp palpation/ or percussion/ or pulse/ or exp vital signs/ 517012

18 exp *Respiratory Tract Infections/ 529915

19 risk factors/ 955289

20 "length of stay"/ or patient admission/ or patient discharge/ or (criteria adj4 discharge).tw. 156283

21 exp intensive care units, pediatric/ or respiratory care units/ or (nicu or icu or picu or intensive-care).tw. 231239

22 morbidity/ or prevalence/ or exp mortality/ 781380

23 exp Diagnostic Imaging/ or radiography, thoracic/ or ((chest adj1 x-ray*) or (chest adj1 xray*)).tw. 2929342

24 exp Hematologic Tests/ or ((blood adj1 test*) or (blood adj1 exam*) or (virologic* adj1 investigation*)).tw. 297077

25 Urinalysis/ 9268

26 Nasal Lavage Fluid/ or ((nose or nasal) adj1 (mucus or mucosa)).tw. 10677

27 Emergency Service, Hospital/ or (emergency adj1 department*).tw. 157373

28 asthma/ or hypersensitivity, immediate/ or (atopy or atopic).tw. 186484

29 (salbutamol or ventolin or levalbuterol or adrenalin* or epinephrin* or (beta* adj2 adrenergic*) or (beta* adj2 agonist*) or ics or (inhaled adj1 corticosteroid*) or montelukast).tw. 133389

30 exp Albuterol/ad, tu 7239

31 exp Epinephrine/ad, tu 10031

32 exp Bronchodilator Agents/ad, tu or bronchodilat*.tw. 63186

33 exp steroids/ad, tu 245236

34 exp Cholinergic Antagonists/ or receptors, adrenergic, beta-2/ or (cholinergic adj1 receptor adj1 block* adj1 agent*).tw. 91309

35 exp Anti-Inflammatory Agents/ 568228

36 exp Adrenal Cortex Hormones/ or (corticosteroid* or (cortico adj1 steroid*) or glucocorticoid* or gluco corticoid*).tw. 513603

37 Leukotriene Antagonists/ or (Leukotriene adj1 receptor adj1 block* adj1 agent*).tw. 3242

38 Saline Solution, Hypertonic/ad, tu or (hypertonic and (saline adj1 solution)).tw. 3206

39 exp Aerosols/ and exp Sodium Chloride/ 410

40 exp "nebulizers and vaporizers"/ and exp sodium chloride/ 116

41 ((aerosoli#ed adj1 saline) or (nebuli#ed adj1 saline)).tw. 203

42 exp Oxygen Inhalation Therapy/ or *Oxygen/ad, st [Administration & Dosage, Standards] 30943

43 (exp Oximetry/ or oximet*.tw.) and (exp "reproducibility of results"/ or (reliability or function or (technical adj1 specification*) or (percutaneous adj1 measurement*)).tw. or exp blood gas analysis/ or ((pulse adj1 oximet*) or (supplementa* adj1 oxygen) or (oxygen adj1 saturation) or (oxygen adj1 therap*) or (oxygen adj1 treatment*)).tw.) 22242

44 continuous positive airway pressure/ or positive pressure respiration/ or (bubble adj1 CPAP).tw. 27198

45 exp Physical Therapy Modalities/ 178123

46 Physical Therapy Specialty/ or physical therapists/ 5873

47 (physiotherap* or (physical adj therap*)).tw. 59298

48 ((nasal* or nose or naso) adj3 suction*).tw. 122

49 suction/ or (deep adj1 suction*).tw. 13138

50 saline.tw. and Administration, Intranasal/ 756

51 ((saline adj1 drop*) or (nasal adj1 saline)).tw. 483

52 exp Fluid Therapy/ or Rehydration Solutions/ 22592

53 enteral nutrition/ or exp parenteral nutrition/ or intubation, gastrointestinal/ 50460

54 (((non adj1 oral) or oral) and (feed* or hydration or fluid* or solution* or therap*)).tw. 211378

55 exp bacterial infections/ 961373

56 exp otitis media/ 25757

57 exp Meningitis/ 58718

58 exp *anti-bacterial agents/tu or (antibiotic* or 3z or aruzilina or atizor or azadose or azasite or azatril or azenil or azibiot or azibiot-neo or azimin or azithral or azithromycin or azitrocin or azitromax or azitromicin or azitromicina or aziwok or azomyne or aztrin or azydrop or azyter or azythromycin or bazyt or cp-62933 or cp-62993 or cp62933 or cp62993 or forcin or inedol or infectoazit or isv-401 or isv401 or kromicin or macrozit or mezatrin or octavax or ordipha or ribotrex or sumamed or sunamed or tobyl or tromix or trozocina or ultreon or vinzam or xithrone or xz-450 or xz450 or zaret or zarom or zetamax or zeto or zibramax or zifin or zimericina or zistic or zithromax or zithrox or zitinn or zitrim or zitrobifan or zitrocin or zitromax or zmax).tw. 490886

59 exp Sepsis/ 141157

60 exp Urinary Tract Infections/ 50669

61 Tracheitis/ 1560

62 (serious adj1 bacterial adj1 infection*).tw. 1294

63 infection control/ or exp primary prevention/ or patient isolation/ 213304

64 COVID-19/ or SARS-CoV-2/ 234537

65 (2019-novel or 2019nCoV or 2019-nCoV or COVID-19 or COVID19 or COVID-2019 or COVID2019 or CONVID-19 or CONVID19 or CORVID-19 or CORVID19 or CoV2 or CoV-2 or HCoV* or Ncov* or Ncorona* or Ncorono* or NcovChina* or NcovChinese* or NcovHubei* or NcovWuhan* or SARS2 or SARS-2 or SARScoronavirus2 or SARScoronavirus-2 or SARScoronovirus2 or SARScoronovirus-2 or SARSCov19 or SARSCov-19 or SARS-CoV-2 or SARSCoV-2 or SARSCoV2 or WN-CoV or WNCoV or wuhan-virus).tw. 341860

66 ((pneumonia* or outbreak* or respiratory-illness* or respiratory-disease* or respiratory-symptom* or seafood-market* or food-market* or wildlife) and (Wuhan or China or Chinese or Hubei or Huanan)).tw. 21170

67 ((new or novel or nouveau or risk factors or "2019" or Wuhan or Hubei or Huanan or China or Chinese) adj3 (coronavirus* or corona virus* or betacoronavirus* or CoV or HCoV)).tw. 75998

68 POST-ACUTE COVID-19 SYNDROME/ or (longCOVID* or postCOVID* or postcoronavirus* or postSARS*).tw. 2221

69 (coronavirus/ or betacoronavirus/ or coronavirus infections/) and (disease outbreaks/ or epidemics/ or pandemics/) 40238

70 ((coronavirus* or corona-virus* or betacoronavirus*) adj3 (pandemic* or epidemic* or outbreak* or crisis)).tw. 15507

71 exp Antibodies, Monoclonal/ or (monoclonal-antibod* or clonal-antibod* or hybridoma-antibod* or nirsevimab or medi-8897 or medi8897 or sp-0232 or sp-232 or sp0232 or sp232 or Motavizumab or medi-524 or medi524 or numax or Palivizumab or abbosynagis or medi-493 or medi493 or synagis or synagys).tw. 362383

72 or/13-71 9536442

73 5 and 72 28221

74 exp pregnant women/ or exp pregnancy/ or prenatal care/ or (parturition or ante-natal or antenatal* or pre-natal* or prenatal* or pregnan*).tw. 1176170

75 exp immunization/ or (immunis* or immuniz* or vaccin*).tw. 554939

76 (Respiratory-syncytial-virus-vaccine* or RSV-vaccine* or Arexvy).tw. 910

77 (74 and 75 and 5) or (74 and 76) 271

78 (75 and 5) or 76 4709

79 12 or 73 or 78 29996

80 (newborn* or new-born* or baby or babies or neonat* or neo-nat* or infan* or toddler* or aged-1 or aged-one or one-year-old or 1-year-old or under-two or under-2 or younger-than-two or younger-than-2 or below-two or below-2 or under-24-months or younger-than-24-months or below-24-months or aged-1-to-23-months or aged-one-to-twenty-three-months).tw,kf,hw. 1692458

81 exp Bronchiolitis Obliterans/ or (bronchiolitis adj1 obliterans).af. 5735

82 (79 and 80) not 81 13211

83 77 not 81 271

84 82 or 83 13251

85 limit 84 to (english language and yr="2000 -Current") 9850

**Embase <1974 to 2023 June 19>**

Search date: 21/06/23

1 bronchiolitis/ or viral bronchiolitis/ or exp human respiratory syncytial virus/ or respiratory syncytial virus infection/ or (bronchiolit* or wheez* or (Respiratory adj1 Syncytial adj1 Virus*)).af. or rsv.tw. 93187

2 limit 1 to (editorial or letter or note) 7059

3 1 and (exp controlled clinical trial/ or exp practice guideline/ or meta analysis/ or "review"/ or "systematic review"/) 16414

4 1 and exp evidence based medicine/ 6467

5 (1 not 2) or 3 or 4 86742

6 *bronchiolitis/co, di, dm, dr, dt, ep, et, pc, rt, th 3796

7 *viral bronchiolitis/co, di, dm, dr, dt, ep, et, pc, th 548

8 6 or 7 4336

9 limit 8 to (editorial or letter or note) 571

10 8 and (exp controlled clinical trial/ or exp practice guideline/ or meta analysis/ or "review"/ or "systematic review"/) 1137

11 8 and exp evidence based medicine/ 555

12 (8 not 9) or 10 or 11 3866

13 History/ or exp Epidemiology/ 4735239

14 reproducibility/ or exp validity/ or scoring system/ or exp reliability/ 847991

15 exp disease severity/ or (severity adj2 illness).tw. 2249656

16 differential diagnosis/ or physical examination/ or palpation/ or percussion/ or blood pressure measurement/ or blood pressure monitoring/ or pulse rate/ or vital sign/ or ascultation.tw. 813575

17 exp *respiratory tract infection/ 235051

18 risk factor/ 1336657

19 "length of stay"/ or hospital admission/ or hospital discharge/ or (criteria adj4 discharge).tw. 638513

20 intensive care unit/ or medical intensive care unit/ or neonatal intensive care unit/ or pediatric intensive care unit/ or (nicu or icu or picu or intensive-care).tw. 452434

21 morbidity/ or prevalence/ or mortality/ or childhood mortality/ or infant mortality/ 2024864

22 exp diagnostic imaging/ or exp thorax radiography/ or ((chest adj1 x-ray*) or (chest adj1 xray*)).tw. 485079

23 exp blood examination/ or ((blood adj1 test*) or (haem* adj1 exam*) or (haem* adj1 test*) or (virologic* adj1 investigation*)).tw. 370894

24 exp urinalysis/ 127990

25 nose mucus/ or nose mucosa/ or (nasal adj1 lavage adj1 fluid*).tw. 18815

26 (emergency adj1 department*).tw. 193543

27 exp asthma/ or atopy/ 315735

28 (salbutamol or ventolin or levalbuterol or adrenalin* or epinephrin* or (beta* adj2 adrenergic*) or (beta* adj2 agonist*) or ics or (inhaled adj1 corticosteroid*) or montelukast).tw. 178202

29 salbutamol/ad, do, dt 18740

30 epinephrine/ad, do, dt 5593

31 exp bronchodilating agent/ad, do, dt or bronchodilat*.tw. 99057

32 exp steroid/ad, do, dt 649505

33 exp cholinergic receptor blocking agent/ or (cholinergic adj1 antagonist*).tw. 318374

34 exp antiinflammatory agent/ 2480733

35 exp corticosteroid/ or (corticosteroid* or (cortico adj1 steroid*) or glucocorticoid* or gluco corticoid*).tw. 1152309

36 exp leukotriene receptor blocking agent/ or (leukotriene adj1 antagonist*).tw. 23668

37 (sodium chloride/ad, do, dt and (aerosol/ or hypertonic solution/ or exp nebulizer/ or vaporizer/)) or (hypertonic and (saline adj1 solution)).tw. 1308

38 ((aerosoli#ed adj1 saline) or (nebuli#ed adj1 saline)).tw. 284

39 exp oximetry/ or exp oxygen therapy/ or oxygen/ad, do 124833

40 (exp oximetry/ or oximet*.tw.) and (reproducibility/ or (reliability or function or (technical adj1 specification*) or (percutaneous adj1 measurement*)).tw. or exp blood gas analysis/ or ((pulse adj1 oximet*) or (supplementa* adj1 oxygen) or (oxygen adj1 saturation) or (oxygen adj1 therap*) or (oxygen adj1 treatment*)).tw.) 24303

41 positive end expiratory pressure ventilation/ or ((continuous adj1 positive adj1 airway adj1 pressure) or (positive adj1 pressure adj1 respiration) or (bubble adj1 CPAP)).tw. 22005

42 exp physiotherapy/ 107415

43 (physiotherap* or physical therap*).tw. 96671

44 ((nasal* or nose or naso) adj3 suction*).tw. 183

45 suction/ or (deep adj1 suction*).tw. 12931

46 sodium chloride/na [Intranasal Drug Administration] 177

47 ((saline adj1 drop*) or (nasal adj1 saline)).tw. 667

48 enteric feeding/ or exp parenteral nutrition/ or exp digestive tract intubation/ 92632

49 (((non adj1 oral) or oral) and (feed* or hydration or fluid* or solution* or therap*)).tw. 337435

50 exp fluid therapy/ or oral rehydration solution/ 112397

51 exp bacterial infection/ 955606

52 exp otitis media/ 38079

53 exp meningitis/ 114158

54 exp *antiinfective agent/dt or (antibiotic* or 3z or aruzilina or atizor or azadose or azasite or azatril or azenil or azibiot or azibiot-neo or azimin or azithral or azithromycin or azitrocin or azitromax or azitromicin or azitromicina or aziwok or azomyne or aztrin or azydrop or azyter or azythromycin or bazyt or cp-62933 or cp-62993 or cp62933 or cp62993 or forcin or inedol or infectoazit or isv-401 or isv401 or kromicin or macrozit or mezatrin or octavax or ordipha or ribotrex or sumamed or sunamed or tobyl or tromix or trozocina or ultreon or vinzam or xithrone or xz-450 or xz450 or zaret or zarom or zetamax or zeto or zibramax or zifin or zimericina or zistic or zithromax or zithrox or zitinn or zitrim or zitrobifan or zitrocin or zitromax or zmax).tw. 977691

55 exp sepsis/ 334492

56 exp urinary tract infection/ 139560

57 exp tracheitis/ 4182

58 (serious adj1 bacterial adj1 infection*).tw. 1891

59 infection control/ or patient care/ or isolation.tw. 787000

60 coronavirus disease 2019/ or experimental coronavirus disease 2019/ 363085

61 (2019-novel or 2019nCoV or 2019-nCoV or COVID-19 or COVID19 or COVID-2019 or COVID2019 or CONVID-19 or CONVID19 or CORVID-19 or CORVID19 or CoV2 or CoV-2 or HCoV* or Ncov* or Ncorona* or Ncorono* or NcovChina* or NcovChinese* or NcovHubei* or NcovWuhan* or SARS2 or SARS-2 or SARScoronavirus2 or SARScoronavirus-2 or SARScoronovirus2 or SARScoronovirus-2 or SARSCov19 or SARSCov-19 or SARS-CoV-2 or SARSCoV-2 or SARSCoV2 or WN-CoV or WNCoV or wuhan-virus).tw. 418425

62 ((pneumonia* or outbreak* or respiratory-illness* or respiratory-disease* or respiratory-symptom* or seafood-market* or food-market* or wildlife) and (Wuhan or China or Chinese or Hubei or Huanan)).tw. 23979

63 ((new or novel or nouveau or ("length of stay" or hospital admission or hospital discharge or (criteria adj4 discharge)) or "2019" or Wuhan or Hubei or Huanan or China or Chinese) adj3 (coronavirus* or corona virus* or betacoronavirus* or CoV or HCoV)).tw. 85970

64 long COVID/ or (longCOVID* or postCOVID* or postcoronavirus* or postSARS*).tw. 4909

65 (coronavirinae/ or betacoronavirus/ or coronavirus infection/) and (epidemic/ or pandemic/) 9531

66 ((coronavirus* or corona-virus* or betacoronavirus*) adj3 (pandemic* or epidemic* or outbreak* or crisis)).tw. 17154

67 severe-acute-respiratory-syndrome-coronavirus-2.hw. 97668

68 coronavirus-disease-2019.hw. 363661

69 exp Monoclonal antibody/ or (monoclonal-antibod* or clonal-antibod* or hybridoma-antibod* or nirsevimab or medi-8897 or medi8897 or sp-0232 or sp-232 or sp0232 or sp232 or Motavizumab or medi-524 or medi524 or numax or Palivizumab or abbosynagis or medi-493 or medi493 or synagis or synagys).tw. 833604

70 or/13-69 14210720

71 5 and 70 69784

72 pregnant woman/ or exp pregnancy/ or prenatal care/ or (parturition or ante-natal or antenatal* or pre-natal* or prenatal* or pregnan*).tw. 1159109

73 exp immunization/ or (immunis* or immuniz* or vaccin*).tw. 656913

74 (Respiratory-syncytial-virus-vaccine* or RSV-vaccine* or Arexvy).tw. 1061

75 (72 and 73 and 5) or (72 and 74) 512

76 (73 and 5) or 74 8119

77 12 or 71 or 76 71881

78 (newborn* or new-born* or baby or babies or neonat* or neo-nat* or infan* or toddler* or aged-1 or aged-one or one-year-old or 1-year-old or under-two or under-2 or younger-than-two or younger-than-2 or below-two or below-2 or under-24-months or younger-than-24-months or below-24-months or aged-1-to-23-months or aged-one-to-twenty-three-months).tw,kf,hw,dq. 1649049

79 bronchiolitis obliterans/ or (bronchiolitis adj1 obliterans).af. 10869

80 (77 and 78) not 79 20025

81 75 not 79 503

82 80 or 81 20142

83 limit 82 to (english language and embase and yr="2000 -Current") 11881

**Cochrane Library**

Search date: 21/06/23

Search Name: Bronchiolitis edited 21.06.23

#1 MeSH descriptor: [Bronchiolitis] explode all trees

#2 bronchiolit* or wheez* or (Respiratory Syncytial Virus*) or rsv

#3 MeSH descriptor: [Respiratory Syncytial Viruses] explode all trees

#4 MeSH descriptor: [Respiratory Syncytial Virus Infections] explode all trees

#5 MeSH descriptor: [Natural History] explode all trees

#6 MeSH descriptor: [Epidemiology] explode all trees

#7 MeSH descriptor: [Severity of Illness Index] explode all trees

#8 MeSH descriptor: [Diagnosis, Differential] explode all trees

#9 MeSH descriptor: [Physical Examination] explode all trees

#10 MeSH descriptor: [Respiratory Tract Infections] explode all trees

#11 MeSH descriptor: [Risk Factors] explode all trees

#12 MeSH descriptor: [Length of Stay] explode all trees

#13 MeSH descriptor: [Patient Admission] explode all trees

#14 MeSH descriptor: [Intensive Care Units] explode all trees

#15 MeSH descriptor: [Morbidity] explode all trees

#16 MeSH descriptor: [Mortality] explode all trees

#17 MeSH descriptor: [Diagnostic Imaging] explode all trees

#18 MeSH descriptor: [Hematologic Tests] explode all trees

#19 MeSH descriptor: [Urinalysis] explode all trees

#20 MeSH descriptor: [Nasal Lavage Fluid] explode all trees

#21 MeSH descriptor: [Emergency Service, Hospital] explode all trees

#22 MeSH descriptor: [Albuterol] explode all trees

#23 MeSH descriptor: [Epinephrine] explode all trees

#24 MeSH descriptor: [Steroids] explode all trees

#25 MeSH descriptor: [Bronchodilator Agents] explode all trees

#26 MeSH descriptor: [Cholinergic Antagonists] explode all trees

#27 MeSH descriptor: [Anti-Inflammatory Agents] explode all trees

#28 MeSH descriptor: [Adrenal Cortex Hormones] explode all trees

#29 MeSH descriptor: [Leukotriene Antagonists] explode all trees

#30 MeSH descriptor: [Saline Solution, Hypertonic] explode all trees

#31 MeSH descriptor: [Aerosols] explode all trees

#32 MeSH descriptor: [Nebulizers and Vaporizers] explode all trees

#33 MeSH descriptor: [Sodium Chloride] explode all trees

#34 (#31 or #32) and #33

#35 MeSH descriptor: [Oxygen Inhalation Therapy] explode all trees

#36 MeSH descriptor: [Oxygen] explode all trees and with qualifier(s): [administration & dosage - AD, standards - ST]

#37 MeSH descriptor: [Oximetry] explode all trees

#38 MeSH descriptor: [Reproducibility of Results] explode all trees

#39 #37 and #38

#40 MeSH descriptor: [Physical Therapy Modalities] explode all trees

#41 MeSH descriptor: [Physical Therapy Specialty] explode all trees

#42 MeSH descriptor: [Suction] explode all trees

#43 MeSH descriptor: [Fluid Therapy] explode all trees

#44 MeSH descriptor: [Infusions, Intravenous] explode all trees

#45 MeSH descriptor: [Administration, Oral] explode all trees

#46 #43 and (#44 or #45)

#47 MeSH descriptor: [Administration, Intranasal] explode all trees

#48 saline (Word variations have been searched)

#49 #48 and #47

#50 MeSH descriptor: [Rehydration Solutions] explode all trees

#51 MeSH descriptor: [Bacterial Infections] explode all trees

#52 MeSH descriptor: [Otitis Media] explode all trees

#53 MeSH descriptor: [Meningitis] explode all trees

#54 MeSH descriptor: [Anti-Bacterial Agents] explode all trees and with qualifier(s): [therapeutic use - TU]

#55 MeSH descriptor: [Sepsis] explode all trees

#56 MeSH descriptor: [Urinary Tract Infections] explode all trees

#57 MeSH descriptor: [Tracheitis] explode all trees

#58 MeSH descriptor: [Radiography, Thoracic] explode all trees

#59 MeSH descriptor: [Asthma] explode all trees

#60 MeSH descriptor: [Hypersensitivity, Immediate] explode all trees

#61 MeSH descriptor: [Receptors, Adrenergic, beta-2] explode all trees

#62 MeSH descriptor: [Continuous Positive Airway Pressure] explode all trees

#63 MeSH descriptor: [Enteral Nutrition] explode all trees

#64 MeSH descriptor: [Parenteral Nutrition] explode all trees

#65 MeSH descriptor: [Intubation, Gastrointestinal] explode all trees

#66 MeSH descriptor: [Infection Control] explode all trees

#67 MeSH descriptor: [Primary Prevention] explode all trees

#68 MeSH descriptor: [Patient Isolation] explode all trees

#69 (History or Epidemiolog* or "severity of illness" or “disease severity” or scoring system* or diagnosis or physical exam* or auscultation or "blood pressure" or palpation* or percussion or pulse or vital sign* or Respiratory Tract Infection* or risk factor* or "length of stay" or admission or discharge or morbidit* or prevalence or mortalit* or "Diagnostic Imaging" or ((chest or thorac*) and (x-ray* or xray* or “x ray” or radiograph*)) or ((Hematolog* or Haematolog* or blood or virolog* or urine) and (Test or tests or exam* or investigation*)) or Urinalys* or “Nasal Lavage” or ((nose or nasal) and (mucosa or mucus)) or emergency department* or asthma* or atopy or atopic or hypersensitiv*) (Word variations have been searched)

#70 salbutamol or albuterol or ventolin or levalbuterol or adrenalin* or epinephrin* or beta2 adrenergic* or beta2 agonist* or ics or corticosteroid* or cortico steroid*OR cortico-steroid* or glucocorticoid* or gluco corticoid* or gluco-corticoid* or montelukast or Bronchodilat* or steroid or steroids or Cholinergic Antagonist* or cholinergic receptor* or Anti Inflammatory Agent* or Adrenal Cortex Hormone* or Leukotriene Antagonist* or Leukotriene receptor* or "Hypertonic Saline" or (("Sodium Chloride" or saline) and (nebuliz* or nebulis* or vaporiz* or vaporis* or aerosol* or intranasal or "intra nasal" or intra-nasal or nasal)) (Word variations have been searched)

#71 (Oxygen or ((Oximetry or oximeter*) and ("reproducibility of results" or reliability or validity or function* or technical specification* or percutaneous measurement* or blood gas analys*)) or CPAP or “continuous positive airway pressure” or “positive pressure respiration” or “positive end respiratory pressure”) (Word variations have been searched)

#72 Physical Therap* or physiotherap* or ((nasal* or nose or naso) and (suction* or toilet or irrigation)) or suction* or saline drop* or "nasal saline" or "intranasal saline" (Word variations have been searched)

#73 (Fluid Therap* or Intravenous infusion* or “non oral” or Rehydrat* or “enteric feeding” or “parenteral nutrition” or “parenteral feeding” or “enteral nutrition” or oral* administ* or bacterial infection* or "otitis media" or Meningitis or antibacterial agent* or anti bacterial agent* or antimicrobial agent* or anti microbial agent* or antibiotic* or 3z or aruzilina or atizor or azadose or azasite or azatril or azenil or azibiot or azibiot-neo or azimin or azithral or azithromycin or azitrocin or azitromax or azitromicin or azitromicina or aziwok or azomyne or aztrin or azydrop or azyter or azythromycin or bazyt or cp-62933 or cp-62993 or cp62933 or cp62993 or forcin or inedol or infectoazit or isv-401 or isv401 or kromicin or macrozit or mezatrin or octavax or ordipha or ribotrex or sumamed or sunamed or tobyl or tromix or trozocina or ultreon or vinzam or xithrone or xz-450 or xz450 or zaret or zarom or zetamax or zeto or zibramax or zifin or zimericina or zistic or zithromax or zithrox or zitinn or zitrim or zitrobifan or zitrocin or zitromax or zmax or Sepsis or septic or Urinary Tract Infection* or tracheitis or serious bacterial infection* or “infection control” or “primary prevention” or isolation or “patient care”) (Word variations have been searched)

#74 “intensive care” or ICU or "respiratory care" or NICU or PICU

#75 MeSH descriptor: [SARS-CoV-2] this term only

#76 MeSH descriptor: [COVID-19] this term only

#77 ((corona* or corono*) NEAR (virus* or viral* or virinae*)) (Word variations have been searched)

#78 (coronavirus* or coronovirus* or coronavirinae* or CoV or CoV2 or CoV-2 or HCoV*) (Word variations have been searched)

#79 (“2019 nCoV” or 2019nCoV or nCoV2019 or nCoV-2019 or COVID-19 or COVID19 or CORVID-19 or CORVID19 or WNCoV or HCoV-19 or HCoV19 or (2019 NEXT novel*) or Ncov or SARSCoV-2 or SARSCoV2 or SARSCov19 or SARSCov-19 or Ncov or Ncorona* or Ncorono* or NcovWuhan* or NcovHubei* or NcovChina* or NcovChinese* or SARS2 or SARS-2 or SARScoronavirus2 or SARScoronavirus-2 or SARScoronovirus2 or SARScoronovirus-2)

#80 (respiratory* NEAR/2 (symptom* or disease* or illness* or condition*) NEAR/10 (Wuhan* or Hubei* or China* or Chinese* or Huanan*))

#81 ((seafood-market* or food-market* or pneumonia*) NEAR/10 (Wuhan* or Hubei* or China* or Chinese* or Huanan*))

#82 ((outbreak* or wildlife* or pandemic* or epidemic*) NEAR (Wuhan* or Hubei or China* or Chinese* or Huanan*))

#83 (COVID-2019 or COVID2019)

#84 MeSH descriptor: [Post-Acute COVID-19 Syndrome] this term only

#85 (longCOVID* or postCOVID* or postcoronavirus* or postSARS*)

#86 MeSH descriptor: [Antibodies, Monoclonal] explode all trees

#87 (monoclonal-antibod* or clonal-antibod* or hybridoma-antibod* or nirsevimab or medi-8897 or medi8897 or sp-0232 or sp-232 or sp0232 or sp232 or Motavizumab or medi-524 or medi524 or numax or Palivizumab or abbosynagis or medi-493 or medi493 or synagis or synagys)

#88 #5 or #6 or #7 or #8 or #9 or #10 or #11 or #12 or #13 or #14 or #15 or #16 or #17 or #18 or #19 or #20 or #21 or #22 or #23 or #24 or #25 or #26 or #27 or #28 or #29 or #30 or #34 or #35 or #36 or #39 or #40 or #41 or #42 or #46 or #49 or #50 or #51 or #52 or #53 or #54 or #55 or #56 or #57 or #58 or #59 or #60 or #61 or #62 or #63 or #64 or #65 or #66 or #67 or #68 or #69 or #70 or #71 or #72 or #73 or #74 or #75 or #76 or #77 or #78 or #79 or #80 or #81 or #82 or #83 or #84 or #85 or #86 or #87

#89 (#1 or #2 or #3 or #4) and #88

#90 MeSH descriptor: [Pregnant Women] explode all trees

#91 MeSH descriptor: [Pregnancy] explode all trees

#92 MeSH descriptor: [Prenatal Care] this term only

#93 (parturition or ante-natal or antenatal* or pre-natal* or prenatal* or pregnan*)

#94 #90 OR #91 or #92 or #93

#95 MeSH descriptor: [Immunization] explode all trees

#96 (immunis* or immuniz* or vaccin*)

#97 #95 OR #96

#98 Respiratory-syncytial-virus-vaccine* or RSV-vaccine* or Arexvy

#99 (#94 and #97 and (#1 or #2 or #3 or #4)) or (#94 and #98)

#100 (#97 and (#1 or #2 or #3 or #4)) or #98

#101 #89 or #100

#102 (newborn* or new born* or baby or babies or neonat* or neo nat* or infan* or toddler* or “aged 1” or “aged one” or “one year old” or “1 year old” or “under two” or “under 2” or “younger than two” or “younger than 2” or “below two” or “below 2” or “under 24 months” or “younger than 24 months” or “below 24 months” or “aged 1 to 23 months” or “aged one to twenty three months”)

#103 MeSH descriptor: [Bronchiolitis Obliterans] explode all trees

#104 ("bronchiolitis obliterans")

#105 (#101 and #102) not (#103 or #104)

#106 #99 not (#103 or #104)

#107 #105 or #106 with Cochrane Library publication date from Jan 2000 to present

**Results = 2164**

**CINAHL**

Search date 21/06/23

S20 S18 AND S19

Limiters - English; Published Date: 20000101-; Peer Reviewed (56)

S19 newborn* or new-born* or baby or babies or neonat* or neo-nat* or infan* or toddler* or aged-1 or aged-one or one-year-old or 1-year-old or under-two or under-2 or younger-than-two or younger-than-2 or below-two or below-2 or under-24-months or younger-than-24-months or below-24-months or aged-1-to-23-months or aged-one-to-twenty-three-months (553,820)

S18 S9 AND S17 (114)

S17 S10 OR S11 OR S12 OR S13 OR S14 OR S15 OR S16 (202,865)

S16 (saline W1 drop*) or (nasal W1 saline) (104)

S15 (MH "Administration, Intranasal") and saline (145)

S14 physiotherap* or "physical therap*" (87,362)

S13 (MH "Physical Therapy+") (159,654)

S12 (MH "Suctioning, Nasopharyngeal") OR (MH "Suction") OR (deep W1 suction*) OR ((nasal* or nose or naso) W3 suction*) (2,755)

S11 (MH "Rehabilitation, Pulmonary")

Limiters - Published Date: 20000101-20011231 (106)

S10 (MH "Chest Physiotherapy (Saba CCC)") OR (MH "Chest Physiotherapy (Iowa NIC)") OR (MH "Chest Physical Therapy+") (870)

S9 (s8 not s5) or s6 or s7 (8,657)

S8 S1 OR S2 OR S3 OR S4 (10,610)

S7 (S1 OR S2 OR S3 OR S4) and (MH "Professional Practice, Evidence-Based+") (108)

S6 S1 OR S2 OR S3 OR S4

Limiters - Publication Type: Clinical Trial, Critical Path, Meta Analysis, Practice Guidelines, Randomized Controlled Trial, Review, Systematic Review (1,501)

S5 S1 OR S2 OR S3 OR S4

Limiters - Publication Type: Case Study, Commentary, editorial, Letter (2,006)

S4 bronchiolit* or wheez* or "respiratory syncytial virus*" or rsv (10,438)

S3 (MH "Respiratory Syncytial Virus Infections") OR (MH "Respiratory Syncytial Viruses") (2,772)

S2 (MH "Bronchial Diseases")

Limiters - Published Date: 20000101-20001231 (40)

S1 (MH "Bronchiolitis+") (2,540)

**PubMed**

Search date 21/6/23

#1 bronchiolitis OR bronchiolitic OR respiratory-syncytial-virus* OR wheez* OR rsv

#2 History OR Epidemiolog* OR "severity of illness" OR "disease severity" OR "scoring system" OR diagnosis OR physical-exam* OR auscultation OR "blood pressure" OR palpation* OR percussion OR pulse OR vital-sign* OR Respiratory-Tract-Infection* OR risk-factor* OR "length of stay" OR admission OR discharge OR "intensive care" OR NICU OR ICU OR PICU OR "respiratory care" OR morbidit* OR prevalence OR mortalit* OR "Diagnostic Imaging" OR ((chest OR thorax OR thoracic) AND (x-ray* OR xray* OR radiograph*)) OR ((Hematolog* OR Haematolog* OR blood OR virolog* OR urine) AND (Test OR tests OR exam* OR investigation*)) OR Urinalys* OR "Nasal Lavage" OR ((nose OR nasal) AND (mucosa OR mucus)) OR emergency-department* OR asthma* OR atopy OR atopic OR hypersensitiv* OR salbutamol OR albuterol OR ventolin OR levalbuterol OR adrenalin* OR epinephrin* OR beta2-adrenergic* OR beta2-agonist* OR ics OR corticosteroid* OR cortico-steroid* OR glucocorticoid* OR gluco corticoid* OR montelukast OR Bronchodilat* OR steroid OR steroids OR Cholinergic-Antagonist* OR cholinergic-receptor* OR Anti-Inflammatory-Agent* OR Adrenal-Cortex-Hormone* OR Leukotriene-Antagonist* OR Leukotriene-receptor* OR "Hypertonic Saline" OR (("Sodium Chloride" OR saline) AND (nebuliz* OR nebulis* OR vaporiz* OR vaporis* OR aerosol* OR intranasal OR intra-nasal OR nasal)) OR Oxygen OR ((Oximetry OR oximeter*) AND ("reproducibility of results" OR reliability OR validity OR function* OR technical-specification* OR percutaneous-measurement* OR blood-gas-analys*)) OR CPAP OR "continuous positive airway pressure" OR "positive pressure respiration" OR "positive end respiratory pressure" OR Physical-Therap* OR physiotherap* OR ((nasal* OR nose OR naso) AND suction*) OR suction* OR saline-drop* OR "nasal saline" OR "nasal toilet" OR "nasal irrigation" OR Fluid-Therap* OR Intravenous-infusion* OR "non oral" OR Rehydrat* OR "enteric feeding" OR "parenteral nutrition" OR "parenteral feeding" OR "enteral nutrition" OR oral* AND administ* OR bacterial-infection* OR "otitis media" OR Meningitis OR antibacterial-agent* OR anti-bacterial-agent* OR antimicrobial-agent* OR anti-microbial-agent* OR antibiotic* OR 3z OR aruzilina OR atizor OR azadose OR azasite OR azatril OR azenil OR azibiot OR azibiot-neo OR azimin OR azithral OR azithromycin OR azitrocin OR azitromax OR azitromicin OR azitromicina OR aziwok OR azomyne OR aztrin OR azydrop OR azyter OR azythromycin OR bazyt OR cp-62933 OR cp-62993 OR cp62933 OR cp62993 OR forcin OR inedol OR infectoazit OR isv-401 OR isv401 OR kromicin OR macrozit OR mezatrin OR octavax OR ordipha OR ribotrex OR sumamed OR sunamed OR tobyl OR tromix OR trozocina OR ultreon OR vinzam OR xithrone OR xz-450 OR xz450 OR zaret OR zarom OR zetamax OR zeto OR zibramax OR zifin OR zimericina OR zistic OR zithromax OR zithrox OR zitinn OR zitrim OR zitrobifan OR zitrocin OR zitromax OR zmax OR Sepsis OR septic OR Urinary-Tract-Infection* OR tracheitis OR serious-bacterial-infection* OR "infection control" OR "primary prevention" OR isolation OR "patient care" OR monoclonal-antibod* OR clonal-antibod* OR hybridoma-antibod* OR nirsevimab OR medi-8897 OR medi8897 OR sp-0232 OR sp-232 OR sp0232 OR sp232 OR Motavizumab OR medi-524 OR medi524 OR numax OR Palivizumab OR abbosynagis OR medi-493 OR medi493 OR synagis OR synagys OR (2019-novel OR 2019nCoV OR 2019-nCoV OR COVID-19 OR COVID19 OR COVID-2019 OR COVID2019 OR CONVID-19 OR CONVID19 OR CORVID-19 OR CORVID19 OR CoV2 OR CoV-2 OR HCoV* OR Ncov* OR Ncorona* OR Ncorono* OR NcovChina* OR NcovChinese* OR NcovHubei* OR NcovWuhan* OR SARS2 OR SARS-2 OR SARScoronavirus2 OR SARScoronavirus-2 OR SARScoronovirus2 OR SARScoronovirus-2 OR SARSCov19 OR SARSCov-19 OR SARS-CoV-2 OR SARSCoV-2 OR SARSCoV2 OR WN-CoV OR WNCoV OR wuhan-virus) OR ((pneumonia* OR outbreak* OR respiratory-illness* OR respiratory-disease* OR respiratory-symptom* OR seafood-market* OR food-market* OR wildlife) AND (Wuhan OR China OR Chinese OR Hubei OR Huanan)) OR ((new OR novel OR nouveau OR 19 OR 2019 OR Wuhan OR Hubei OR Huanan OR China OR Chinese) AND (coronavirus* OR corona virus* OR betacoronavirus* OR CoV OR HCoV)) OR (longCOVID* OR postCOVID* OR postcoronavirus* OR postSARS*) OR ((coronavirus* OR corona-virus* OR betacoronavirus*) AND (pandemic* OR epidemic* OR outbreak* OR crisis))

#3 newborn* OR new-born* OR baby OR babies OR neonat* OR neo-nat* OR infan* OR toddler* OR aged-1 OR aged-one OR one-year-old OR 1-year-old OR under-two OR under-2 OR younger-than-two OR younger-than-2 OR below-two OR below-2 OR under-24-months OR younger-than-24-months OR below-24-months OR aged-1-to-23-months OR aged-one-to-twenty-three-months

#4 NOTNLM

#5 "Bronchiolitis Obliterans"

#6 (#1 AND #2 AND #3 AND #4) NOT #5

Limit 2000 onwards; AND English 2168

[Search name: Cate Wilson 300623 Bronchiolitis AND VOI AND Age AND NLM]

#7 #1 AND #2 AND #3) NOT #5

Limit 2013 onwards; AND English 3576

[Search name: Cate Wilson 300623 Bronchiolitis AND VOI AND Age]

#8 ((bronchiolitis OR bronchiolitic OR respiratory-syncytial-virus* OR wheez* OR rsv) AND (parturition OR ante-natal OR antenatal* OR pre-natal* OR prenatal* OR pregnan*) AND (immunis* OR immuniz* OR vaccin*)) OR ((parturition OR ante-natal OR antenatal* OR pre-natal* OR prenatal* OR pregnan*) AND (Respiratory-syncytial-virus-vaccine* OR RSV-vaccine* OR Arexvy))

#9 (#8 AND #4) NOT #5

Limit 2000 onwards; AND English 160

[Search name: Cate Wilson 300623 Bronchiolitis AND Maternal immunization AND NLM]

#10 #8 NOT #5

Limit 2013 onwards; AND English 271

[Search name: Cate Wilson 300623 Bronchiolitis AND Maternal immunization]

#11 ((bronchiolitis OR bronchiolitic OR respiratory-syncytial-virus* OR wheez* OR rsv) AND (immunis* OR immuniz* OR vaccin*)) OR (Respiratory-syncytial-virus-vaccine* OR RSV-vaccine* OR Arexvy)

#12 (#11 AND #3 AND #4) NOT #5

Limit 2000 onwards; AND English 1087

[Search name: Cate Wilson 300623 Bronchiolitis AND Infant immunization AND Age AND NLM]

#13 (#11 AND #3) NOT #5

Limit 2013 onwards; AND English 1725

[Search name: Cate Wilson 300623 Bronchiolitis AND Infant immunization AND Age]

#14 #6 OR #7 OR #9 OR #10 OR #12 OR #13 4384

**Example search strategy for backdated new topics**

**PubMed**

Search date 30/6/23

[Bronchiolitis + ICU +Age) NOT Obliterans]

Filters applied: English, from 2010 - 2014

((bronchiolitis OR bronchiolitic OR respiratory-syncytial-virus* OR wheez* OR rsv) AND ("intensive care" OR NICU OR ICU OR PICU OR "respiratory care") AND (newborn* OR new-born* OR baby OR babies OR neonat* OR neo-nat* OR infan* OR toddler* OR aged-1 OR aged-one OR one-year-old OR 1-year-old OR under-two OR under-2 OR younger-than-two OR younger-than-2 OR below-two OR below-2 OR under-24-months OR younger-than-24-months OR below-24-months OR aged-1-to-23-months OR aged-one-to-twenty-three-months)) NOT ("Bronchiolitis Obliterans")

[Bronchiolitis + Monoclonal antibodies +Age) NOT Obliterans]

Filters applied: English, from 2010 - 2014

((((bronchiolitis OR bronchiolitic OR respiratory-syncytial-virus* OR wheez* OR rsv) AND (monoclonal-antibod* or clonal-antibod* or hybridoma-antibod* or nirsevimab or medi-8897 or medi8897 or sp-0232 or sp-232 or sp0232 or sp232 or Motavizumab or medi-524 or medi524 or numax or Palivizumab or abbosynagis or medi-493 or medi493 or synagis or synagys) AND (newborn* OR new-born* OR baby OR babies OR neonat* OR neo-nat* OR infan* OR toddler* OR aged-1 OR aged-one OR one-year-old OR 1-year-old OR under-two OR under-2 OR younger-than-two OR younger-than-2 OR below-two OR below-2 OR under-24-months OR younger-than-24-months OR below-24-months OR aged-1-to-23-months OR aged-one-to-twenty-three-months)) NOT ("Bronchiolitis Obliterans")) AND ((2010:2018[pdat]) AND (english[Filter])))

[Bronchiolitis + Maternal immunization) NOT Obliterans]

Filters applied: English, from 2010 - 2014

(((((bronchiolitis OR bronchiolitic OR respiratory-syncytial-virus* OR wheez* OR rsv) AND (parturition OR ante-natal OR antenatal* OR pre-natal* OR prenatal* OR pregnan*) AND (immunis* OR immuniz* OR vaccin*)) OR ((parturition OR ante-natal OR antenatal* OR pre-natal* OR prenatal* OR pregnan*) AND (Respiratory-syncytial-virus-vaccine* OR RSV-vaccine* OR Arexvy))) NOT ("Bronchiolitis Obliterans")) AND ((2010:2018[pdat]) AND (english[Filter])))

[Bronchiolitis + Infant immunization) NOT Obliterans]

Filters applied: English, from 2010 - 2014

((((((bronchiolitis OR bronchiolitic OR respiratory-syncytial-virus* OR wheez* OR rsv) AND (immunis* OR immuniz* OR vaccin*)) OR (Respiratory-syncytial-virus-vaccine* OR RSV-vaccine* OR Arexvy)) AND (newborn* OR new-born* OR baby OR babies OR neonat* OR neo-nat* OR infan* OR toddler* OR aged-1 OR aged-one OR one-year-old OR 1-year-old OR under-two OR under-2 OR younger-than-two OR younger-than-2 OR below-two OR below-2 OR under-24-months OR younger-than-24-months OR below-24-months OR aged-1-to-23-months OR aged-one-to-twenty-three-months)) NOT ("Bronchiolitis Obliterans")) AND ((2010:2018[pdat]) AND (english[Filter])))
